# Postprandial plasma GLP-1 levels are elevated in individuals with postprandial hypoglycaemia following Roux-en-Y gastric bypass – a systematic review

**DOI:** 10.1007/s11154-023-09823-3

**Published:** 2023-07-13

**Authors:** Ryan Joseph Jalleh, Mahesh Michael Umapathysivam, Mark Philip Plummer, Adam Deane, Karen Louise Jones, Michael Horowitz

**Affiliations:** 1 Adelaide Medical School, The University of Adelaide, South Australia, Australia; 2 Endocrine and Metabolic Unit, Royal Adelaide Hospital, South Australia, Australia; 3 Diabetes and Endocrine Services, Northern Adelaide Local Health Network, South Australia, Australia; 4 Intensive Care Unit, Royal Melbourne Hospital, Parkville, VIC Australia

**Keywords:** Bariatric surgery, Glucagon-like peptide-1, Hypoglycaemia, Incretin, Metabolic surgery, Postprandial

## Abstract

**Background and aims**: Bariatric surgery is the most effective treatment in individuals with obesity to achieve remission of type 2 diabetes. Post-bariatric surgery hypoglycaemia occurs frequently, and management remains suboptimal, because of a poor understanding of the underlying pathophysiology. The glucoregulatory hormone responses to nutrients in individuals with and without post-bariatric surgery hypoglycaemia have not been systematically examined. **Materials and methods**: The study protocol was prospectively registered with PROSPERO. PubMed, EMBASE, Web of Science and the Cochrane databases were searched for publications between January 1990 and November 2021 using MeSH terms related to post-bariatric surgery hypoglycaemia. Studies were included if they evaluated individuals with post-bariatric surgery hypoglycaemia and included measurements of plasma glucagon-like peptide-1 (GLP-1), glucose-dependent insulinotropic polypeptide (GIP), insulin, C-peptide and/or glucagon concentrations following an ingested nutrient load. Glycated haemoglobin (HbA_1c_) was also evaluated. A random-effects meta-analysis was performed, and Hedges’ *g* (standardised mean difference) and 95% confidence intervals were reported for all outcomes where sufficient studies were available. The τ^2^ estimate and I^2^ statistic were used as tests for heterogeneity and a funnel plot with the Egger regression-based test was used to evaluate for publication bias. **Results**: From 377 identified publications, 12 were included in the analysis. In all 12 studies, the type of bariatric surgery was Roux-en-Y gastric bypass (RYGB). Comparing individuals with and without post-bariatric surgery hypoglycaemia following an ingested nutrient load, the standardised mean difference in peak GLP-1 was 0.57 (95% CI, 0.32, 0.82), peak GIP 0.05 (-0.26, 0.36), peak insulin 0.84 (0.44, 1.23), peak C-peptide 0.69 (0.28, 1.1) and peak glucagon 0.05 (-0.26, 0.36). HbA_1c_ was less in individuals with hypoglycaemia − 0.40 (-0.67, -0.12). There was no evidence of substantial heterogeneity in any outcome except for peak insulin: τ^2^ = 0.2, I^2^ = 54.3. No publication bias was evident. **Conclusion**: Following RYGB, postprandial peak plasma GLP-1, insulin and C-peptide concentrations are greater in individuals with post-bariatric surgery hypoglycaemia, while HbA_1c_ is less. These observations support the concept that antagonism of GLP-1 would prove beneficial in the management of individuals with hypoglycaemia following RYGB.

PROSPERO Registration Number: CRD42021287515.

**Supplementary Information:**

The online version contains supplementary material available at 10.1007/s11154-023-09823-3.

## Introduction

### Impact of bariatric surgery on obesity, type 2 diabetes and glucagon-like peptide-1 secretion

Bariatric surgery is the most effective treatment in the management of morbid obesity [1]. Roux-en-Y gastric bypass (RYGB) and sleeve gastrectomy are frequently performed procedures and, in general, have favorable short- and longer-term outcomes, including the remission of type 2 diabetes [2]. The exaggerated post-prandial secretion of glucagon-like peptide-1 (GLP-1) and consequent increase in insulin secretion/sensitivity is thought to be central to this effect [3]. The increase in GLP-1 is multifactorial and likely to reflect more rapid gastric pouch emptying and increased delivery of nutrients to the GLP-1-secreting L-cells in the distal small intestine, changes in the bile acid profile to stimulate GLP-1 release mediated via the G-protein-coupled bile acid receptor and alterations in the gut microbiota leading to stimulation of GLP-1 secretion via metabolites [4, 5]. An improvement in glucose tolerance is evident within a few days after surgery and precedes weight loss [3]. Although it is well-recognised that nutrient-induced GLP-1 secretion is greatly increased following some forms of bariatric surgery, it is not clear if individuals who experience hypoglycaemia post-bariatric surgery have a greater GLP-1 response than individuals without hypoglycaemia.

## Prevalence of post-bariatric surgery hypoglycaemia

Post-bariatric surgery hypoglycaemia (PBH) is a complication of metabolic surgery and defined as having a low blood glucose level (< 3.0 mmol/L) associated with autonomic or neuroglycopenic symptoms, although there is no consensus for the threshold glucose level [6]. It occurs in about a quarter of patients following RYGB, characteristically 1–3 h following a meal [7, 8] and has also been described following sleeve gastrectomy [7]. PBH causes substantial morbidity [7, 9]. and, in extreme cases, is life-threatening [10]. The role of GLP-1 in PBH remains unclear with inconsistent observations [11, 12]. Clarification of this is important as GLP-1 receptor antagonists are being evaluated as a potential therapy in early clinical studies [13], where paradoxically, GLP-1 receptor agonists have also been suggested to have benefit in PBH [14]. There is currently no accepted standard medical treatment for PBH. Accordingly, this study has systematically reviewed the literature to evaluate if nutrient-induced peak GLP-1 concentrations are greater in individuals with PBH compared with individuals who have had bariatric surgery but do not have PBH.

### Methods

#### Study design and registration

This systematic review and meta-analysis of cohort and case-control studies was designed in accordance with the latest methodological guidance [15, 16], and was reported in compliance with the Meta-analysis Of Observational Studies in Epidemiology (MOOSE) guidelines [17]. Protocol details were prospectively registered on PROSPERO (CRD42021287515); there were no major protocol deviations.

### Eligibility criteria

We included original research studies that reported a prognostic association between bariatric surgery (Roux-en-Y gastric bypass, sleeve gastrectomy or single anastomosis gastric bypass) and hypoglycaemia. We excluded abstracts and conference presentations, case reports, case series, editorials, expert opinions, publications with incompletely reported data, studies published in language other than English and non-human studies.

### Search strategy

We searched PubMed, EMBASE, Web of Science and Cochrane Database of Systematic Reviews from inception to 27 Nov 2021. Our search strategy included a comprehensive set of relevant search terms (Supplemental 1) and was designed with the support of a professional librarian, experienced in systematic reviews [18].

### Study selection

Two authors (R.J.J. and M.M.U.) independently screened titles and abstracts for potentially relevant studies. The full texts of shortlisted studies were extracted and were assessed against eligibility criteria independently and in duplicate. A third author (M.P.P.) adjudicated any disagreements. We also reviewed the reference and citation lists of included studies for additional potentially relevant studies.

### Data extraction and management

Two authors (R.J.J. and M.M.U.) independently used standardised spreadsheets to extract data from included studies. Where reported, the following were recorded: study design, population baseline characteristics, operative details, diabetes status at the time of the study, test meal contents and definition of hypoglycaemia. The primary outcome was post-bariatric surgery nutrient stimulated peak plasma levels of GLP-1 in individuals with and without hypoglycaemia. Secondary outcomes included (i) post-bariatric surgery nutrient stimulated plasma levels of glucose dependent insulinotropic polypeptide (GIP), insulin and C-peptide in patients with and without hypoglycaemia, (ii) HbA1c, (iii) hypoglycaemic counter-regulatory hormones (cortisol, glucagon, adrenaline, noradrenaline) and (iv) gastric emptying data.

### Risk of bias (quality) assessment

The same authors (R.J.J. and M.M.U.) independently assessed the risk of bias using the Newcastle-Ottawa Scale (NOS). Disagreements in assessment were discussed and consensus obtained.

### Statistical analysis and data synthesis

In studies where outcomes of interest were presented in tables, but not reported in numerical form, the corresponding authors were contacted via email to request this information. No responses were received. The software PlotDigitizer.exe (Huwaldt JA. Plot Digitizer. Version 2.6.9, Free Software Foundation 2020) was used to extract peak concentrations of relevant enteropancreatic hormones and standardized deviations. We tabulated weighted mean differences (reported as Hedges’ *g*) and 95% confidence interval from each study and generated summary estimates using random effects modelling [19]. We performed separate meta-analyses for each outcome where reporting was sufficient across studies; otherwise, we performed qualitative analyses. Random-effects meta-analysis was used to account for potential between-study heterogeneity, and REML (restricted maximum likelihood) was used for the random effect estimation.Statistical heterogeneity is reported as the I^2^ statistic and ${\tau }^{2}$ estimate [20]. Where there were fewer than 10 included studies reporting on an outcome, publication bias was unable to be formally assessed [21]. All data analyses were performed in consultation with a professional biostatistician using STATA (StataCorp. 2021. Stata Statistical Software: Release 17. College Station, TX: StataCorp LLC).

### Search results

**Fig. 1:**
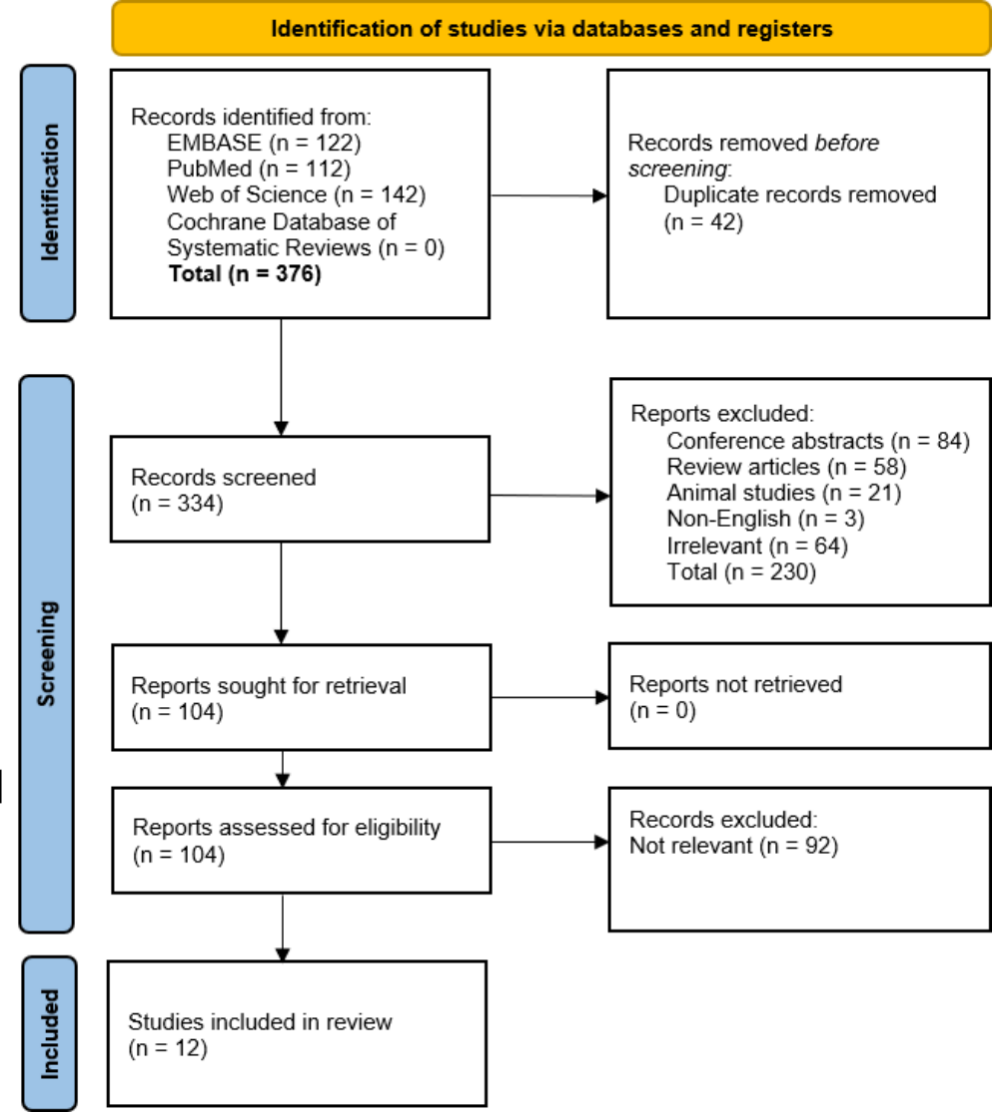
Identification of studies via databases and registers

The search returned 376 results. No additional citations were identified from secondary searching of reference lists. After de-duplication, 334 studies underwent title and abstract screening. 104 potentially relevant studies underwent full-text review, from which 12 studies were included in this review. (Fig. 1). The majority of these 104 studies described an increase in GLP-1 following RYGB (which has been well established) but did not determine whether GLP-1 secretion is increased to a greater extent in individuals with PBH and were, therefore, excluded.

### Description of included studies

Twelve studies involving 324 participants post-bariatric surgery were included. Detailed characteristics of included studies are presented in Table 1. (Table 1)

**Table 1:** Detailed characteristics of included studies (n = 12)

Study	Design	Sample size	Surgery	Age (years ± SEM)	Sex (M/F)	Diabetes (diabetes/remission/no prior diabetes)	Definition of hypoglycaemia	Test meal	Outcome on incretin hormones
Goldfine	Case-control	21	RYGB	48 ± 3	4/17	0/0/21	Prior documented severe hypoglycaemia (severe neuroglycopenic symptoms attributable to hypoglycaemia requiring bystander assistance)	Ensure 240 ml (40 g carbohydrate, 6 g fat, 9 g protein)	Higher fasting and postprandial GLP-1 but lower postprandial GIP in hypoglycaemia group.
Guarino	Cohort	35	RYGB	52 ± 2	9/24	9/26/0	Plasma glucose ≤ 3.3 mmol/L following a standard meal with autonomic/neuroglycopenic symptoms	75 g glucose drink	Comparable postprandial GLP-1 response. GIP not measured.
Kellogg	Cohort	14	RYGB	46 ± 3	2/10 (2 not reported)	1/0/13	Plasma glucose < 3.9 mmol/L following a high carbohydrate meal with autonomic/neuroglycopenic symptoms	High carbohydrate meal (405 kcal, 79% carbohydrate, 11% fat, 10% protein)	Higher postprandial insulin following high carbohydrate meal but GLP-1 and GIP not measured.
Laurenius	Case-control	16	RYGB	47 ± 2	5/11	0/1/15	Prior documented severe hypoglycaemia	Liquid mixed meal (99.5 g carbohydrate, 0.1 g fat, 5.7 g protein)	Comparable postprandial GLP-1 response. GIP not measured.
Lobato	Cohort	23	RYGB	43 ± 3	4/19	0/3/20	Plasma glucose ≤ 3.05 mmol/L following a standard liquid mixed meal with autonomic/neuroglycopenic symptoms	Fresubin 200ml, (300 kcal, 50% carbohydrate, 35% fat, 15% protein)	Higher peak postprandial GLP-1 but no difference in GIP.
Poitou	Cohort	20	RYGB	44 ± 3	4/16	N/A	Plasma glucose ≤ 3 mmol/L following a standard liquid mixed meal with autonomic/neuroglycopenic symptoms	Fresubin 400 ml (800 kcal, 45% carbohydrate, 35% fat, 20% protein)	Comparable postprandial GLP-1 and GIP responses.
Salehi 2014a	Case-control	65	RYGB	48 ± 2	10/55	16/16/33	History of autonomic/neuroglycopenic symptoms following a meal	Ensure Plus, 237 ml (350 kcal, 57% carbohydrate, 28% fat, 15% protein)	Comparable postprandial GLP-1 and GIP responses.
Salehi 2014b	Case-control	16	RYGB	46 ± 3	4/12	4/4/8	Plasma glucose < 2.8 mmol/L following a standard liquid mixed meal with neuroglycopenic symptoms	Ensure Plus, 237 ml (350 kcal, 57% carbohydrate, 28% fat, 15% protein	Trend towards a higher postprandial GLP-1 in the hypoglycaemia group but comparable GIP responses.
Salehi 2019	Case-control	14	RYGB	45 ± 3	3/11	1/1/12	Plasma glucose < 2.8 mmol/L following a standard liquid mixed meal with neuroglycopenic symptoms	Ensure Plus, 237 ml (350 kcal, 57% carbohydrate, 28% fat, 15% protein	Higher postprandial GLP-1 in the hypoglycaemia group but comparable GIP response.
Soeby	Case-control	26	RYGB	43 ± 2	5/21	0/3/23	Plasma glucose ≤ 3.5 mmol/L following a 50 g glucose drink with autonomic/neuroglycopenic symptoms	Fresubin 200 ml, (300 kcal, 50% carbohydrate, 35% fat, 15% protein)	Comparable postprandial GLP-1 and GIP responses.
Tharakan	Cohort	28	RYGB	46 ± 2	9/19	N/A	Prior history of hypoglycaemia fulfilling Whipple’s triad	Ensure Plus	Higher postprandial GLP-1 in the hypoglycaemia group but comparable GIP response.
Vaurs	Cohort	46	RYGB	43 ± 3	N/A	N/A	Plasma glucose < 2.8 mmol/L following a 75 g glucose drink with autonomic/neuroglycopenic symptoms	75 g glucose drink	Comparable postprandial GLP-1 response. GIP not measured.

### Methodological quality

The studies included were observational in design and of small sample sizes associated with large confidence intervals. The studies included were mostly at a low risk of bias as assessed by the Newcastle-Ottawa scale. Results are summarized in Table 2 below. (Table 2).

**Table 2:** Risk of bias assessment by Newcastle-Ottawa scale. Studies with total score 8 or greater (n = 11/12) are considered to be at low risk of bias

Study	Selection (Maximum: ★★★★)	Comparability (Maximum: ★★)	Outcome (Maximum: ★★★)	Total
Goldfine	★★★	★★	★★★	8/9
Guarino	★★★★	★★	★★★	9/9
Kellogg	★★★	★★	★★★	8/9
Laurenius	★★★★	★★	★★★	9/9
Lobato	★★★	★★	★★★	8/9
Poitou	★★★★	★	★★★	8/9
Salehi 2014a	★★★★	★★	★★★	9/9
Salehi 2014b	★★★	★★	★★★	8/9
Salehi 2019	★★★★	★★	★★★	9/9
Soeby	★★★	★★	★★★	8/9
Tharakan	★★	★★	★★★	7/9
Vaurs	★★★★	★★	★★★	9/9

### Outcomes

#### Peak postprandial GLP-1

**Fig. 2:**
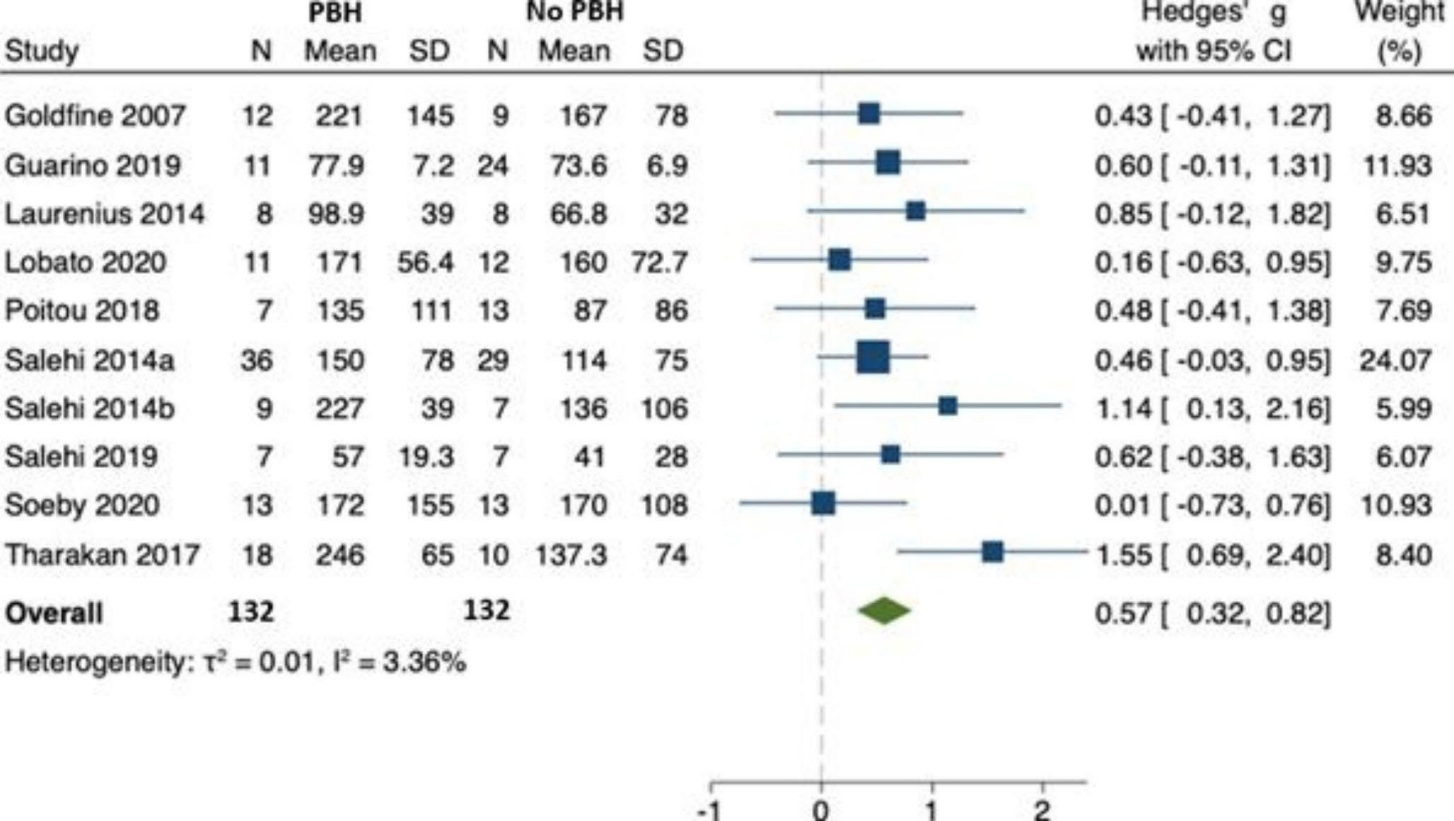
Peak postprandial GLP-1 concentration in those with PBH (n = 132) compared to those without (n = 132)

**Fig. 3:**
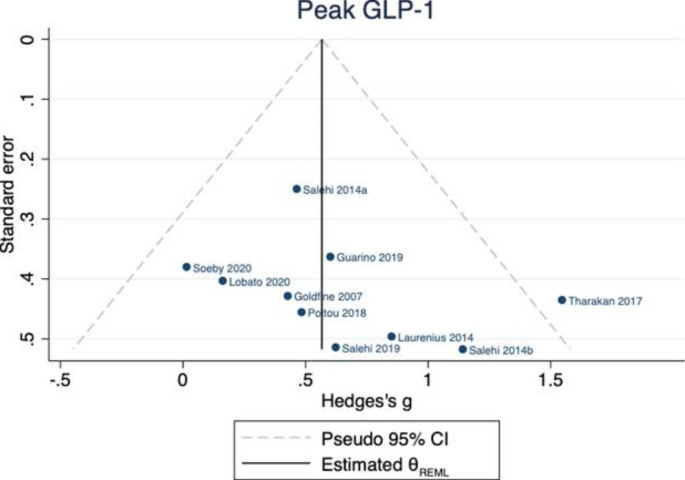
Funnel plot for peak GLP-1 publication bias

In ten studies [22–31] (involving 264 individuals), there was an increase in peak postprandial GLP-1 in those with PBH compared to those without; Hedges’ *g* 0.57 (95% CI 0.32, 0.82) reported this outcome for both PBH (n = 132) and non-PBH (n = 132) cohorts (Fig. 2). There was no evidence of between-study heterogeneity (${\tau }^{2}$=0.01, ${I}^{2}$= 3.36). Only two studies [23, 27] reported the outcome for GLP-1 AUC in both PBH and non-PBH cohorts, hence a meta-analysis for this outcome was not performed. The *p* value for Egger’s test was 0.306. There was no evidence of bias in the funnel plot. (Fig. 3)

### Peak postprandial GIP

**Fig. 4:**
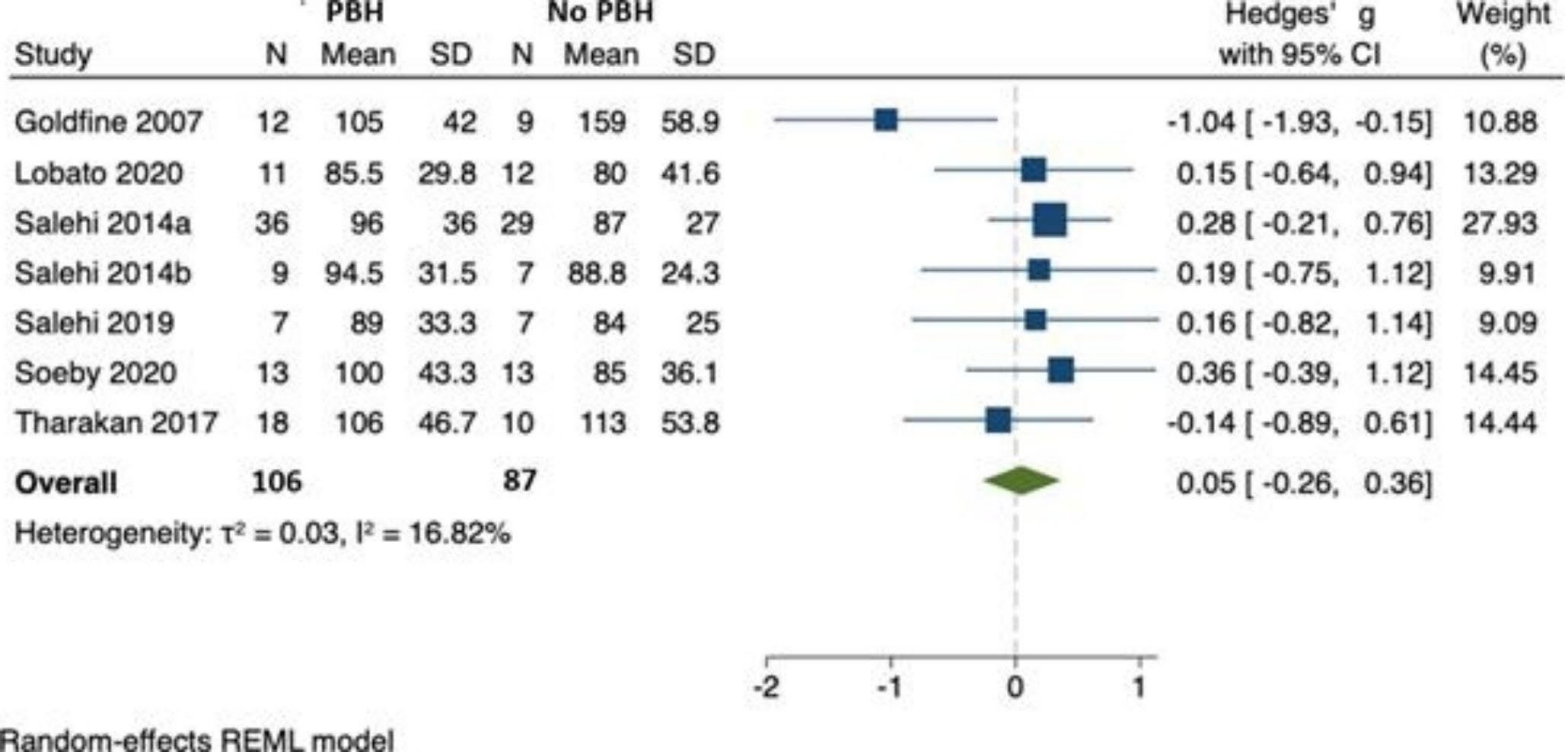
Peak postprandial GIP concentration in those with PBH (n = 106) compared to those without (n = 87)

In 7 studies, involving 193 individuals, there was no difference between postprandial GIP in those with (n = 106) and without (n = 87) PBH [22, 25, 27–31]. (Fig. 4) Hedges’ *g* was 0.05 (95% CI -0.26, 0.36) and there was no evidence of between-study heterogeneity (${\tau }^{2}$=0.03, ${I}^{2}$= 16.82). No study reported GIP AUC. As there were fewer than ten studies, we were unable to formally assess for publication bias.

### Peak postprandial insulin

**Fig. 5:**
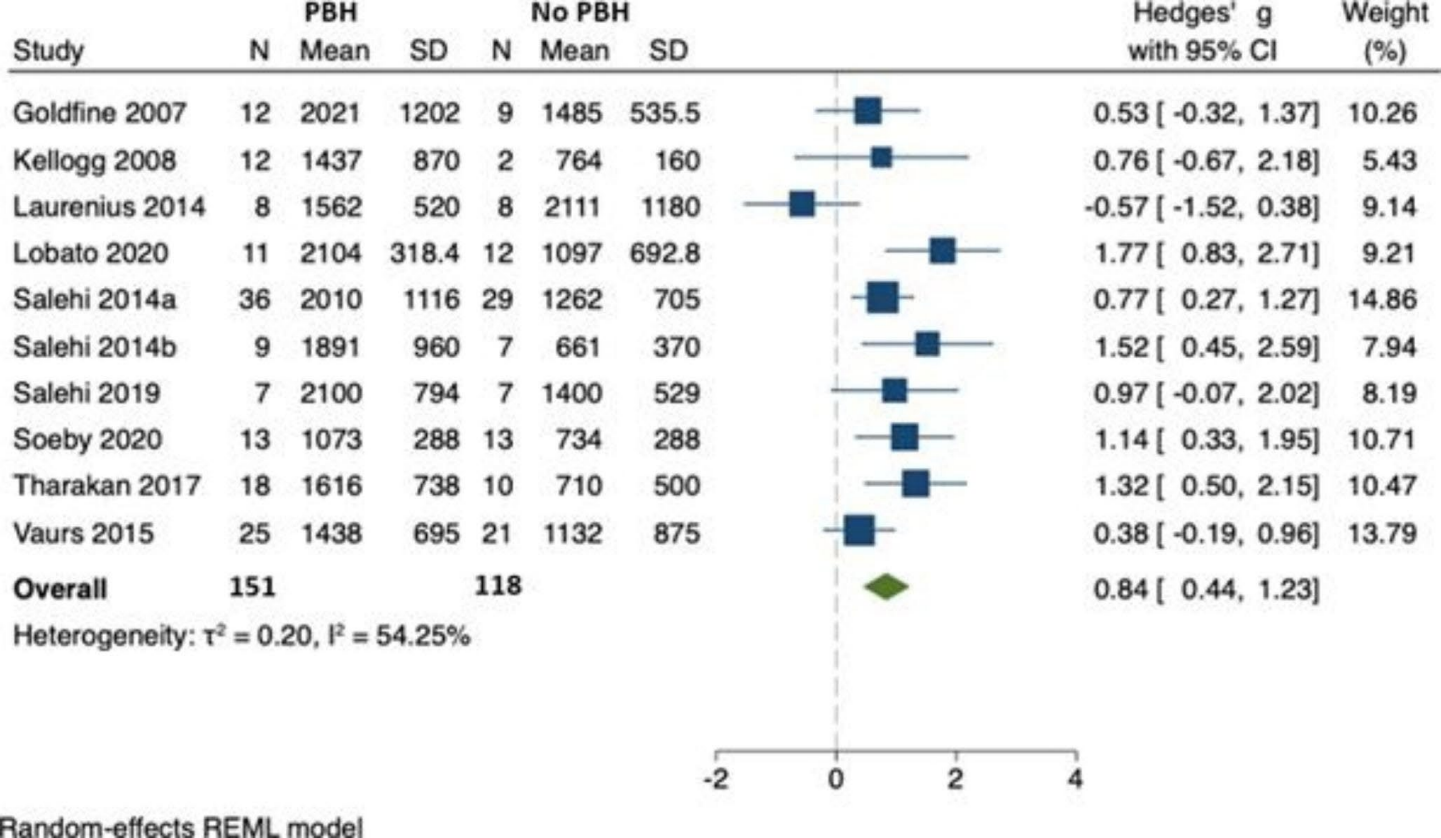
Peak postprandial insulin concentration in those with PBH (n = 132) compared to those without (n = 132)

**Fig. 6:**
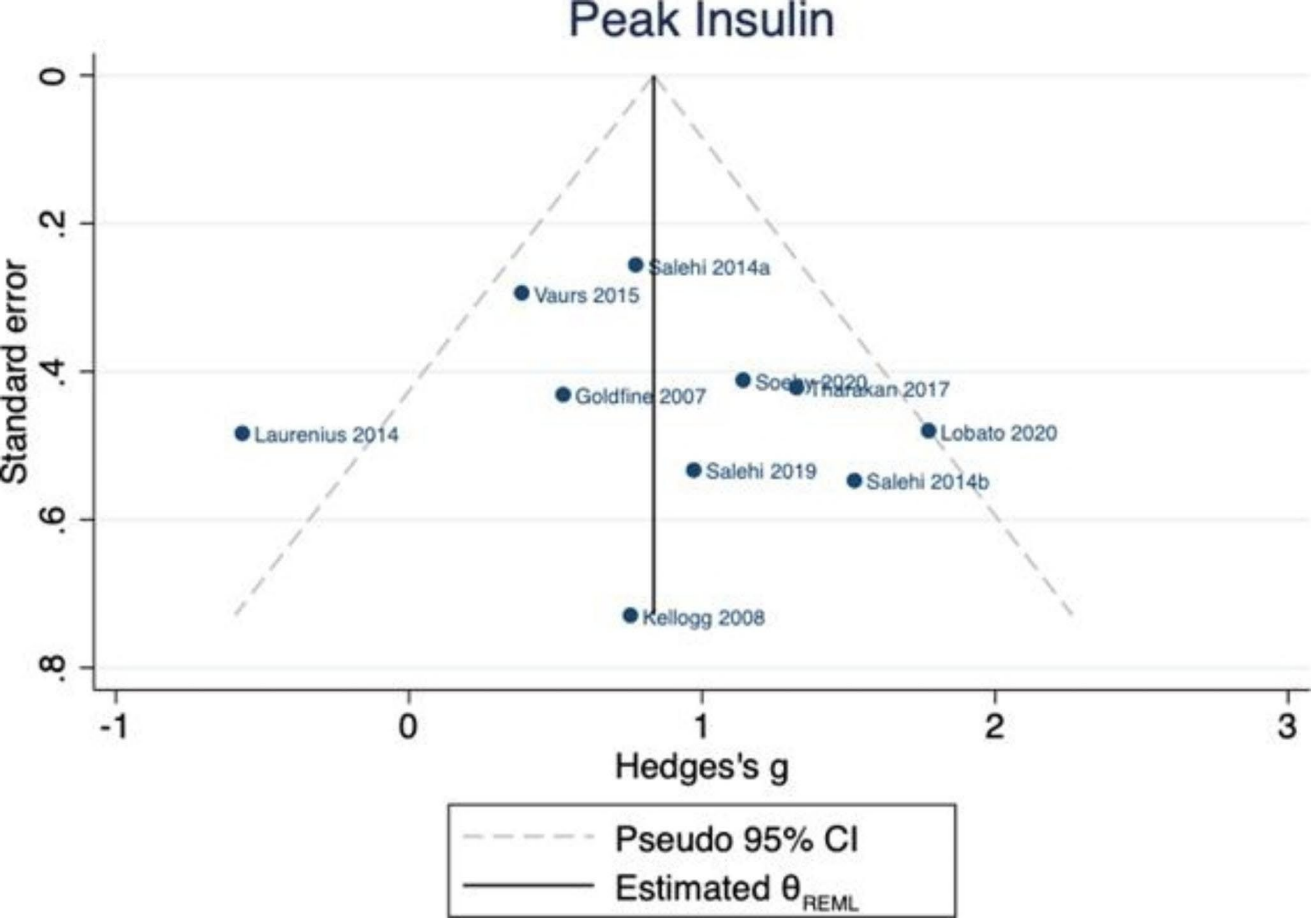
Funnel plot for peak insulin publication bias

In 10 studies (involving 269 individuals), there was an increase in peak postprandial insulin in those with PBH (n = 151) compared to those without (n = 118) [22, 24, 25, 27–33]. Hedges’ *g* was 0.84 (95% CI 0.44, 1.23), however, there was some indication of between-study heterogeneity (${\tau }^{2}$=0.20, ${I}^{2}$= 54.25, p=0.03). One study [24] reported the means, but not standard deviations, of the insulin AUC. This study was not included in the meta-analysis. (Fig. 5) The *p* value for Egger’s test was 0.632. There was no evidence of bias in the funnel plot. (Fig. 6)

### Peak post-prandial C-peptide

**Fig. 7:**
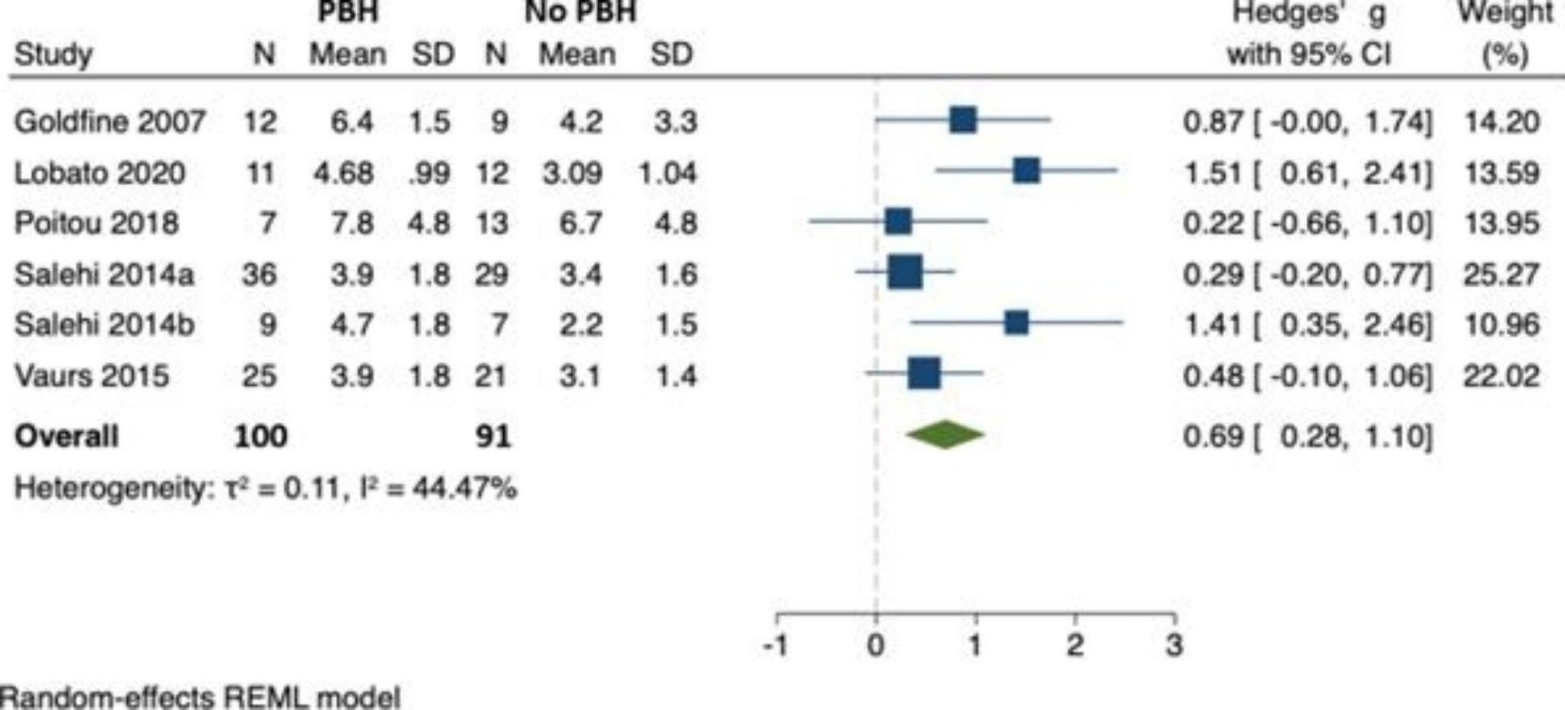
Peak postprandial C-peptide concentration in those with PBH (n = 100) compared to those without (n = 91)

In 6 studies (involving 191 individuals), there was an increase in peak postprandial C-peptide in those with PBH (n = 100) compared to those without (n = 91) [22, 25–28, 33]. Hedges’ *g* was 0.69 (95% CI 0.28, 1.10) and there was no evidence of between-study heterogeneity (${\tau }^{2}$=0.11, ${I}^{2}$= 44.47) (Fig. 7). No study reported the C-peptide AUC. As there were fewer than ten studies, we were unable to formally assess for publication bias.

### Peak glucagon

**Fig. 8:**
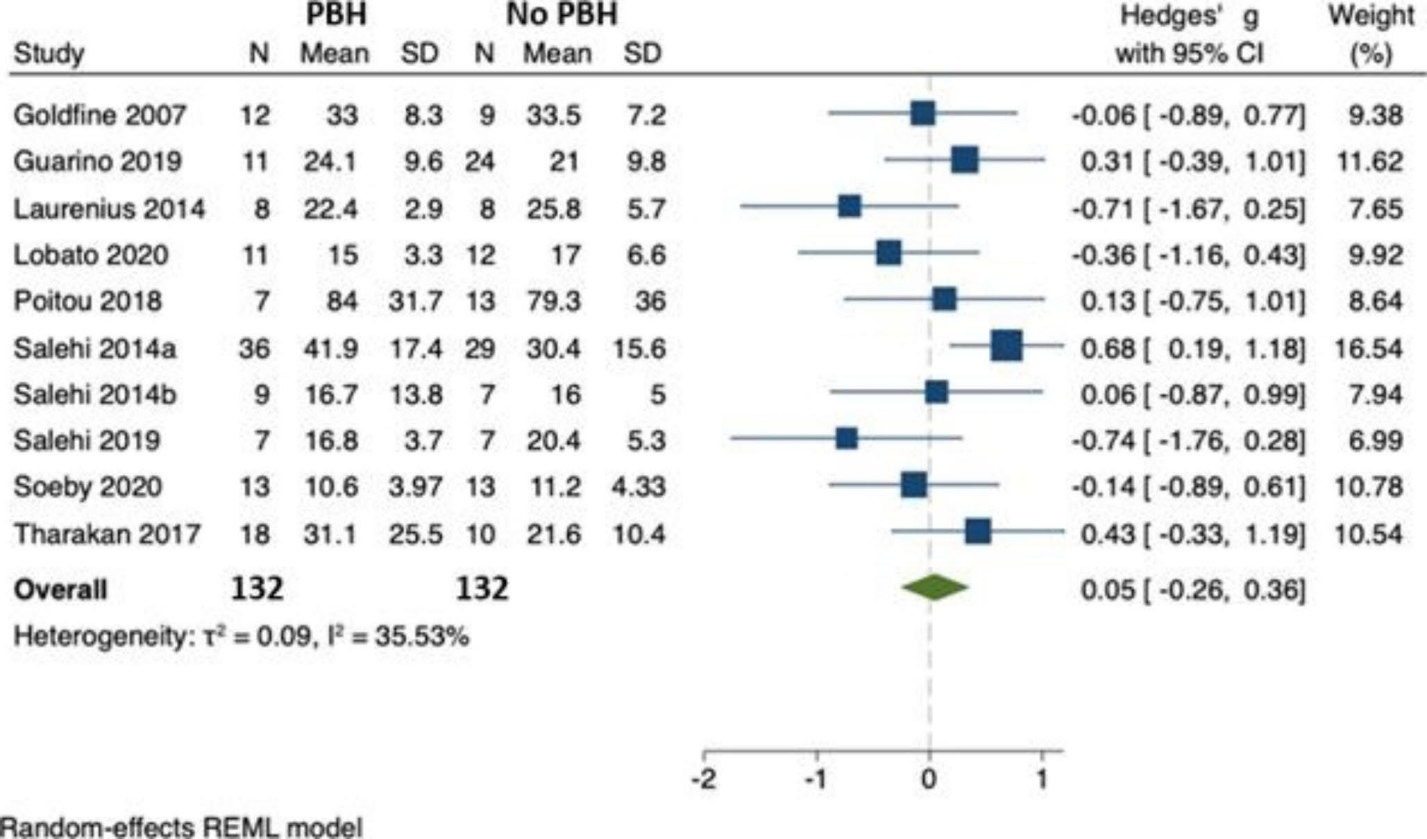
Peak postprandial glucagon concentration in those with PBH (n = 132) compared to those without (n = 132)

**Fig. 9:**
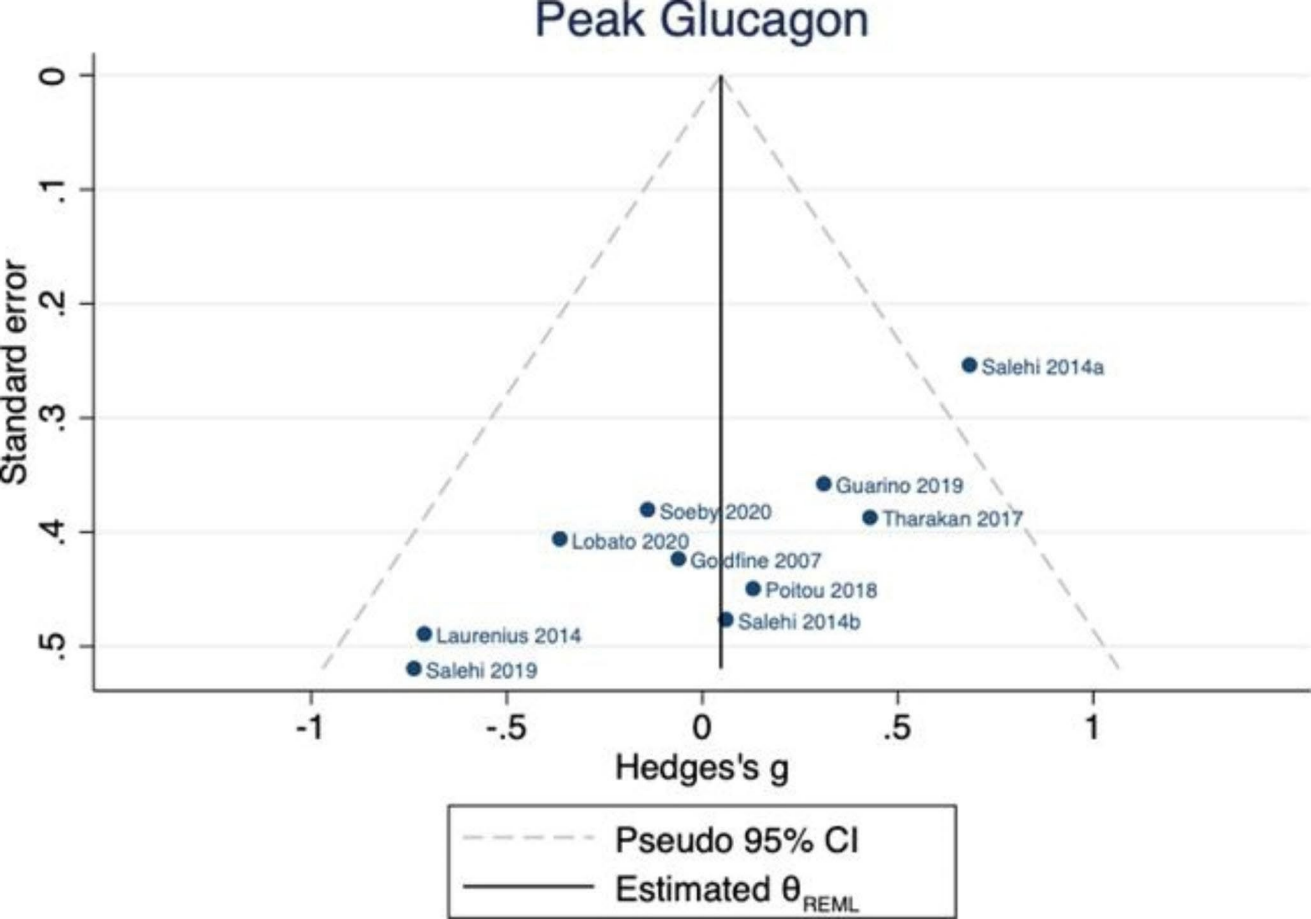
Funnel plot for peak glucagon publication bias

In 10 studies, involving 264 individuals, there was no difference between postprandial glucagon in those with (n = 132) and without (n = 132) PBH [22–31]. Hedges’ *g* was 0.05 (95% CI -0.26, 0.36) and there was no evidence of substantial between-study heterogeneity (${\tau }^{2}$=0.09, ${I}^{2}$= 35.53) (Fig. 8). No study reported the glucagon AUC. The p value for Egger’s test was 0.015. There was no evidence of bias in the funnel plot. (Fig. 9)

### HbA1c

**Fig. 10:**
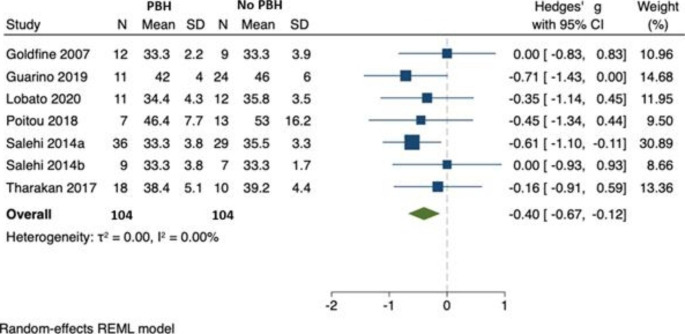
HbA1c in those with PBH (n = 104) compared to those without (n = 104)

In 7 studies, involving 208 individuals, HbA1c was lower in individuals with PBH (n = 104) compared with those without PBH (n = 104) [22, 23, 25–28, 31]. Hedges’ *g* was − 0.40 (95% CI -0.67, -0.12) and there was no evidence of between-study heterogeneity (${\tau }^{2}$=0.00, ${I}^{2}$= 0.00). (Fig. 10) As there were fewer than ten studies, we were unable to formally assess for publication bias.

### Other parameters

The studies were screened for other outcomes, including gastric emptying data (not reported in any study), catecholamines (not reported in any study), cortisol (not reported in any study) insulin secretion (reported in 1 study), disposition index (reported in 1 study) and HOMA-IR (not reported in any study), for which meta-analyses were, accordingly, unable to be performed.

## Discussion

This is the first systematic review to evaluate associations between nutrient-stimulated enterohormones in individuals with postprandial hypoglycaemia following RYGB. Individuals who had post-bariatric surgery hypoglycaemia had greater postprandial GLP-1 and insulin concentrations, but GIP responses were not different, when compared with individuals who had the same type of bariatric surgery but who did not develop hypoglycaemia. Furthermore, in individuals with post-bariatric surgery hypoglycaemia, mean HbA1c was less. There was no substantial between-study heterogeneity in the outcomes analysed apart from peak insulin, where there was a strong association with PBH. The observed heterogeneity is likely due to reflect different frequencies of diabetes and remission as summarized in Table 1. Based on the funnel plots and Egger’s test, there was no evidence of publication bias, apart from the analysis of peak glucagon where the funnel plot was skewed to the right by the largest study [27]. Overall, the pooled results from all studies indicated that there was no association between peak glucagon and PBH. The majority of the studies (11/12) were well designed with a low risk of bias, as evaluated with the Newcastle-Ottawa Scale. Pre-specified sensitivity analyses of low risk of bias studies (included in Supplementary material) did not alter the results.

This meta-analysis of all studies has made it evident that a greater peak GLP-1 response is associated with PBH following RYGB while peak GIP is not. The latter is not surprising given that GIP-secreting K-cells are predominantly found in the duodenum/proximal small intestine which is bypassed following RYGB. There was only one study which found that a greater postprandial peak in GIP was associated with a lower risk of hypoglycaemia [22]. It should be appreciated that this study was published much earlier (2007) than the other studies, and the difference in GIP may be related to variations in surgical technique such as the length of proximal intestine bypassed, however, this information was not provided. Both peak insulin and C-peptide were predictably strongly associated with PBH, consistent with the concept that PBH is driven by exaggerated endogenous insulin secretion. In contrast, there was no evidence of an association between peak glucagon and PBH. We had hypothesized that, reflecting the capacity of GLP-1 to suppress glucagon, that a lower peak glucagon may be associated with PBH. It is possible that PBH is mainly driven by the exaggerated GLP-1 and insulin response rather than suppression of glucagon. As glucagon is released as a counter-regulatory response to hypoglycaemia [34], this may over-ride the suppressive effect of GLP-1 which occurs in a glucose-dependent manner. A lower HbA1c was associated with an increased risk of PBH. This may be explained by either the effect of hypoglycaemia to lower mean blood glucose or a protective effect against hypoglycaemia due to greater insulin resistance associated with higher HbA1c.

The underlying cause of PBH remains unclear, however, there are several putative mechanisms including increased beta cell mass [35], increased insulin-independent glucose uptake [36], increased secretion of GLP-1 [37], altered bile acid metabolism [38], altered gut microbiota [39] and increased fibroblast growth factor-19 [40]. Although GLP-1RA therapy in T2D without concomitant administration of insulin is not associated with hypoglycaemia, there are multiple physiologic changes that occur following RYGB that could explain why hypoglycaemia may occur in this group. RYGB is associated with gastric pouch emptying rates of up to 100 kcal/min [5] compared with gastric emptying rates of 1–4 kcal/min in health, which may lead to a transient marked postprandial glycaemic excursion [41]. The rapid and increased delivery of nutrients to the small intestine is thought to underlie the supraphysiologic levels of GLP-1 [5, 42]. GLP-1 has glucose-dependent effects to augment insulin and inhibit glucagon secretion [43], with a consequent marked glucose-lowering effect. RYGB also alters bile acid composition and the activation of intestinal farnesoid X receptor (FXR) by bile acids increases fibroblast growth factor-19 (FGF-19), an intestinally derived hormone, which reduces hepatic glucose production and increases peripheral glucose disposal independent of insulin [40, 44]. Interestingly, increased FGF-19 levels have been associated with PBH; moreover, the progressive increase in FGF-19 levels over time corresponds to the time course of PBH [40]. Changes to the gut microbiota occur also within 3 months post-RYGB, are sustained in the longer-term [45], and have been associated with lower postprandial glucose levels in rodent models [46]. While these changes are thought to be pivotal to remission of type 2 diabetes, it is likely that, in combination, they also contribute to PBH and in such individuals, we have now shown that GLP-1 responses are greater.

### Limitations

Although all forms of bariatric surgery were included in the search criteria, only studies involving RYGB were identified. Thus, it is not known if there are similar associations between glucoregulatory hormones and PBH with other bariatric surgery procedures such as sleeve gastrectomy or the one anastomosis gastric bypass. Only English language articles were included, however, only 3 identified articles were non-English language. Only peak hormone levels were consistently reported among studies. Data were collected for area under the curve (AUC) hormone levels over time but due to varying time-frames and most studies not reporting this, a meta-analysis could not be performed. There is no standardized meal for the evaluation of PBH and accordingly, there was significant variation in the meals between studies. Similarly, there was varied definitions for hypoglycaemia between studies and we were unable to assess the relationship between severity of postprandial hypoglycaemia and GLP-1. Furthermore, we were unable to evaluate the temporal relationship between GLP-1 and hypoglycaemia/hyperinsulinaemia. The final number of studies included for GIP, C-peptide and HbA1c were too small to analyse for publication bias.

#### Implications and areas for further evaluation

The outcomes of this analysis support an association between the stimulation of GLP-1 with PBH. Early clinical trials have suggested that GLP-1 antagonism [37, 47] may represent a novel therapy for PBH. In a proof-of-principle double-blinded, placebo-controlled, crossover study, the intravenous infusion of exendin (9–39), a GLP-1 receptor antagonist, prevented hypoglycaemia in 8 out of 8 participants with PBH [37]. Our findings support the development of larger-scale clinical trials to evaluate the potential role of GLP-1 antagonism in PBH management. Recent trials have also suggested a role for GLP-1 receptor agonist (RA) treatment as an adjunct to increase rates of type 2 diabetes remission [48]. The effect of GLP-1RAs on PBH has not been established, however, two small, uncontrolled studies reported improvement of PBH following GLP-RA treatment [14, 49] but sample sizes were small (n = 5 and n = 13 respectively). Possible explanations to account for this paradox include slowing of intestinal transit, resulting in decreased stimulation of L-cells [50] and marked suppression of endogenous GLP-1 secretion [51].

Gastric emptying has been recognized as a major determinant of postprandial glycaemic excursions in individuals with and without type 2 diabetes [52] but was not evaluated in any of the included studies. There is no information about the role of gastric emptying/small intestinal transit in the pathogenesis of PBH and this would be of considerable interest. Additionally, there is evidence that an acceleration of gastric emptying is important in the counter-regulation of hypoglycaemia [53]. Catecholamines are an important counter-regulatory mechanism in hypoglycaemia and this response may be blunted following RYGB [54]. It is not known if individuals with PBH have a greater reduction in the catecholamine response to hypoglycaemia.

Finally, our findings suggest additional attention to PBH be given to individuals who have a lower HbA1c as they may be at greater risk.

## Conclusion

Following RYGB, nutrient-induced peak concentrations of GLP-1, insulin and C-peptide are greater, while HbA1c is less in individuals with post-bariatric surgery hypoglycaemia. These observations suggest that antagonism of GLP-1 represents a rational intervention to prevent hypoglycaemia in patients who suffer from post-bariatric surgery hypoglycaemia after RYGB. Further evaluation of the role of gastric emptying and catecholamine response in this condition is warranted.

### Electronic supplementary material

Below is the link to the electronic supplementary material.

Supplementary Material 1

Supplementary Material 2

Supplementary Material 3

## Abbreviations

GIP: Glucose dependent insulinotropic polypeptide
GLP-1: Glucagon-like peptide-1
HbA1c: Haemoglobin A1c
MOOSE: Meta-analysis Of Observational Studies in Epidemiology
NOS: Newcastle-Ottawa Scale
PBH: Post-bariatric surgery hypoglycaemia
RA: Receptor agonist
RYGB: Roux-en-Y gastric bypass
