# Supplementary material for: Postprandial plasma GLP-1 levels are elevated in individuals with postprandial hypoglycaemia following Roux-en-Y gastric bypass – a systematic review
Source: Rev Endocr Metab Disord. 2023 Jul 13;24(6):1075–88. doi: 10.1007/s11154-023-09823-3 (PMC10697890; doi:10.1007/s11154-023-09823-3)
Supplement: Supplementary file 1 — Supplementary Material 1 [file 11154_2023_9823_MOESM1_ESM.docx]

**Supplemental 1**

Search strategy for PubMed:

1. "bariatric surgery"[majr]
2. bariatric surg*[tiab]
3. post bariatric[tiab]
4. gastric bypass[tiab]
5. RYGB[tiab]
6. "roux-en-Y"[tiab]
7. 1 OR 2 OR 3 OR 4 OR 5 OR 6
8. hypoglycemia[majr]
9. hypoglycaemi*[tiab]
10. hypoglycemi*[tiab]
11. 8 OR 9 OR 10
12. incretin[majr]
13. incretin[tiab]
14. “gut hormone”[tiab]
15. glucagon[tiab]
16. “insulinotropic polypeptide”[tiab]
17. 12 OR 13 OR 14 OR 15 OR 16
18. 7 AND 11 AND 17

This PubMed search strategy was reformatted to search Embase, Web of Science and the Cochrane Central Register of Controlled Trials.
