# Supplementary material for: Postprandial plasma GLP-1 levels are elevated in individuals with postprandial hypoglycaemia following Roux-en-Y gastric bypass – a systematic review
Source: Rev Endocr Metab Disord. 2023 Jul 13;24(6):1075–88. doi: 10.1007/s11154-023-09823-3 (PMC10697890; doi:10.1007/s11154-023-09823-3)

Below are the results of a sensitivity analyses that excluded the study (n = 1) with a Newcastle-Ottawa Scale of 7 or less (considered to be at risk of bias). The overall outcomes were unchanged by the sensitivity analyses.


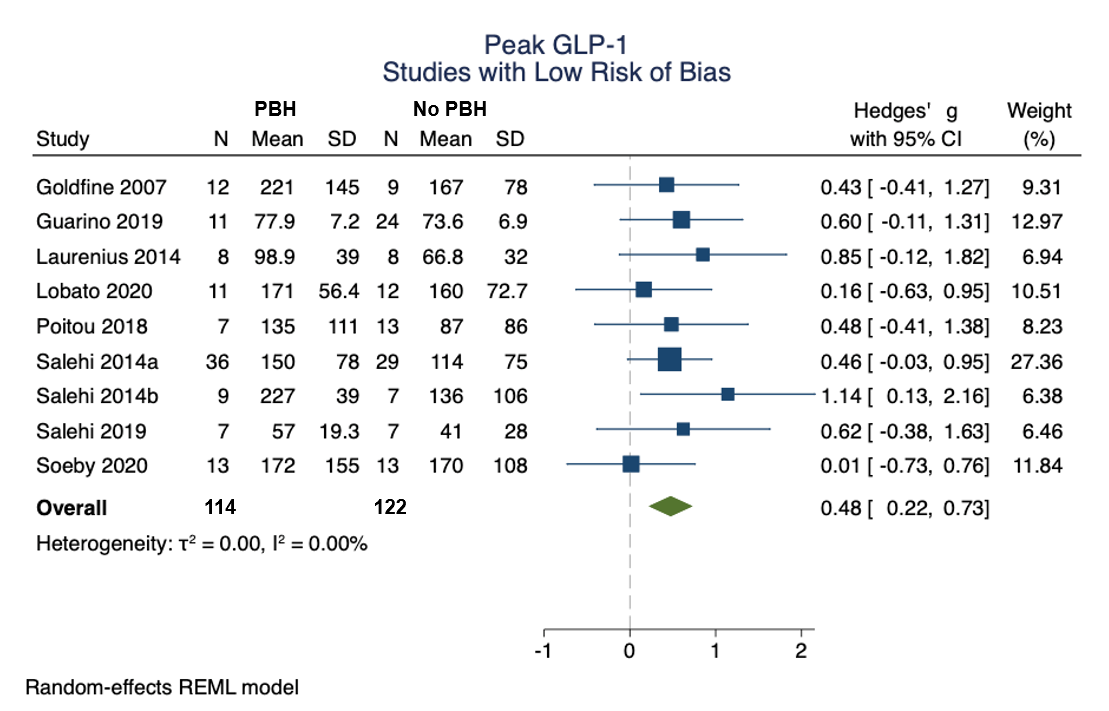


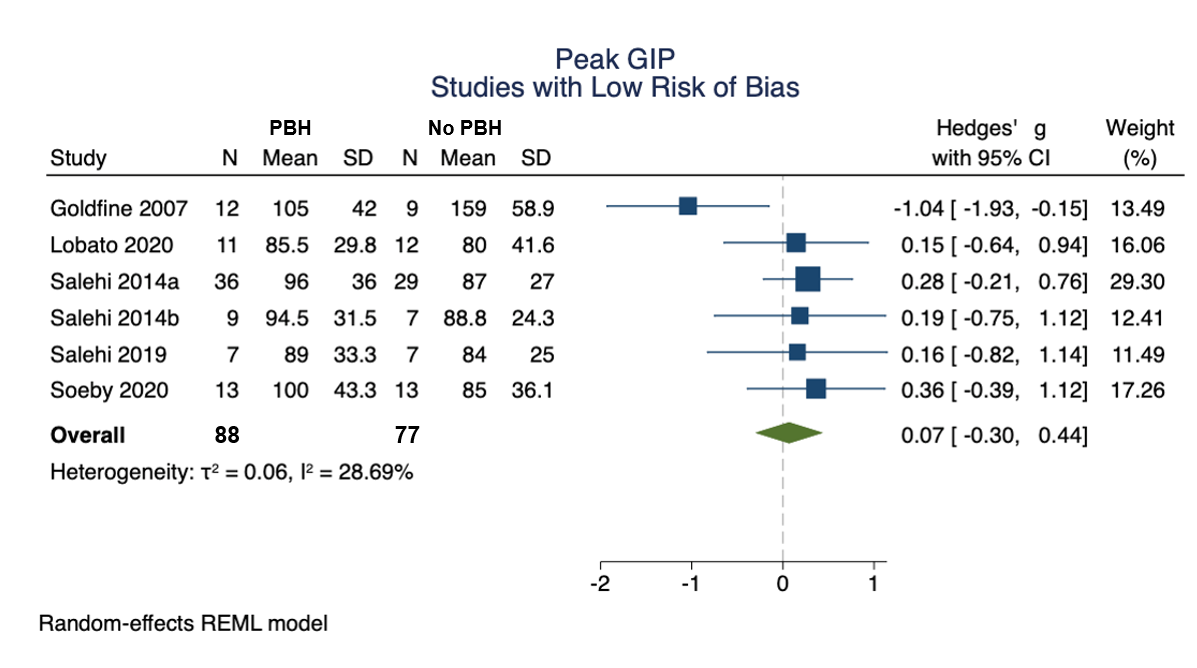


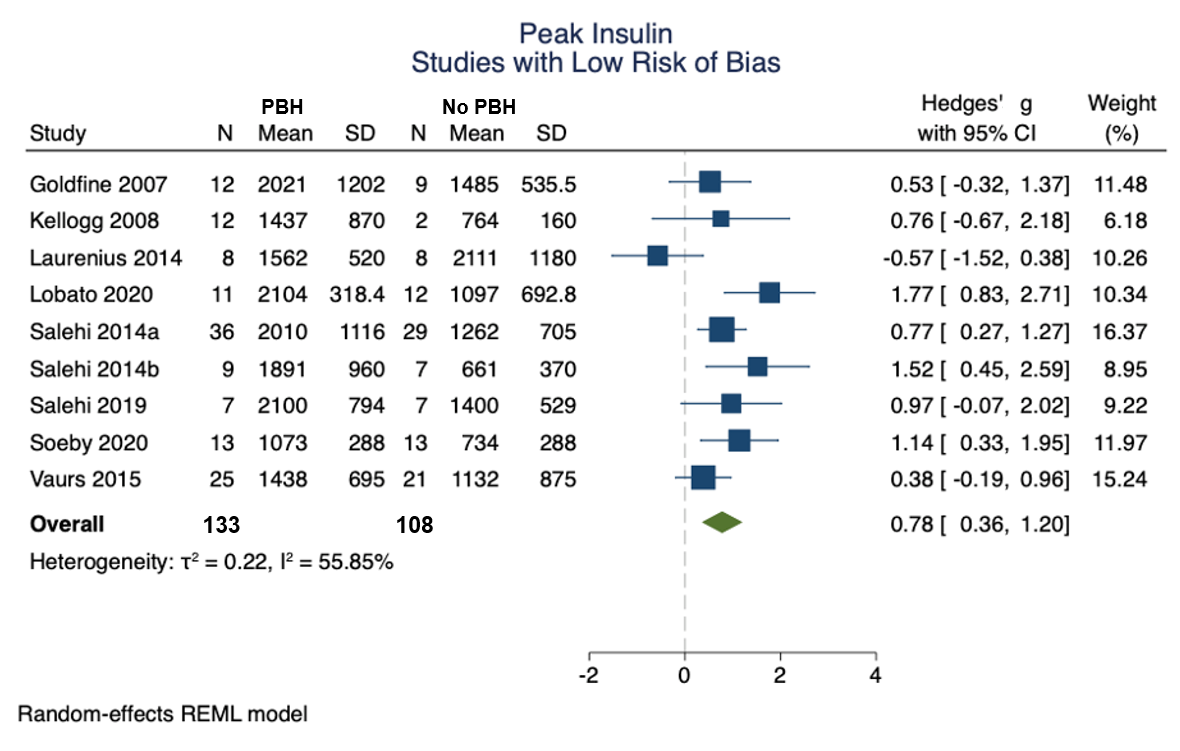


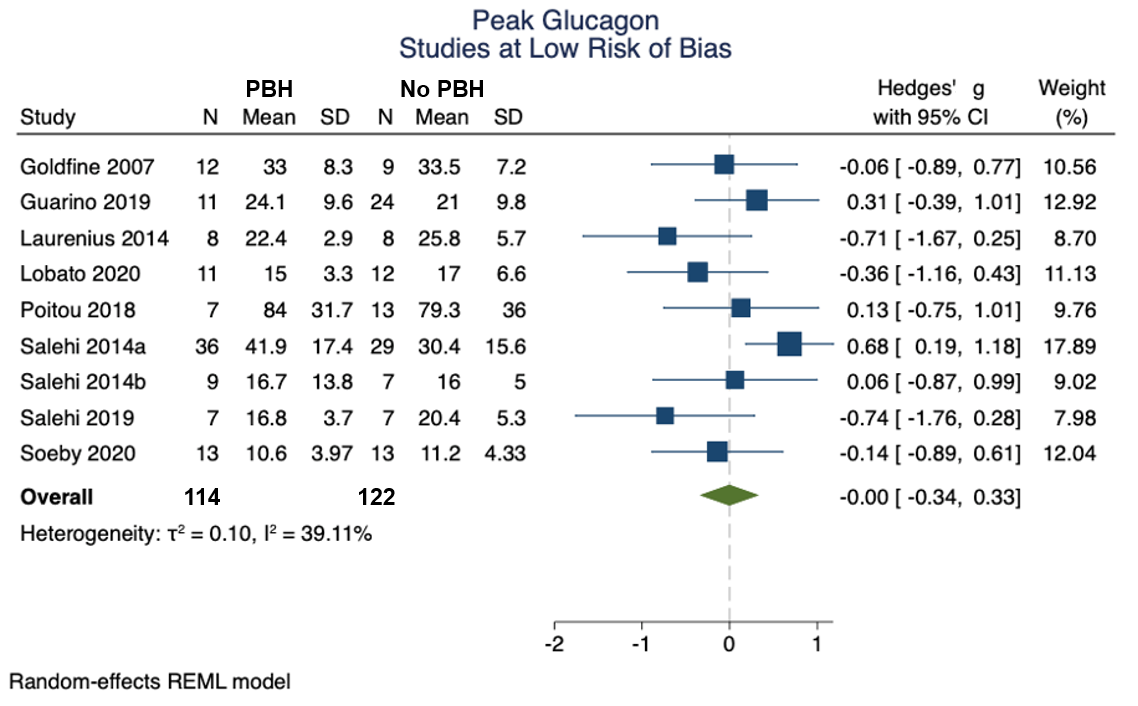


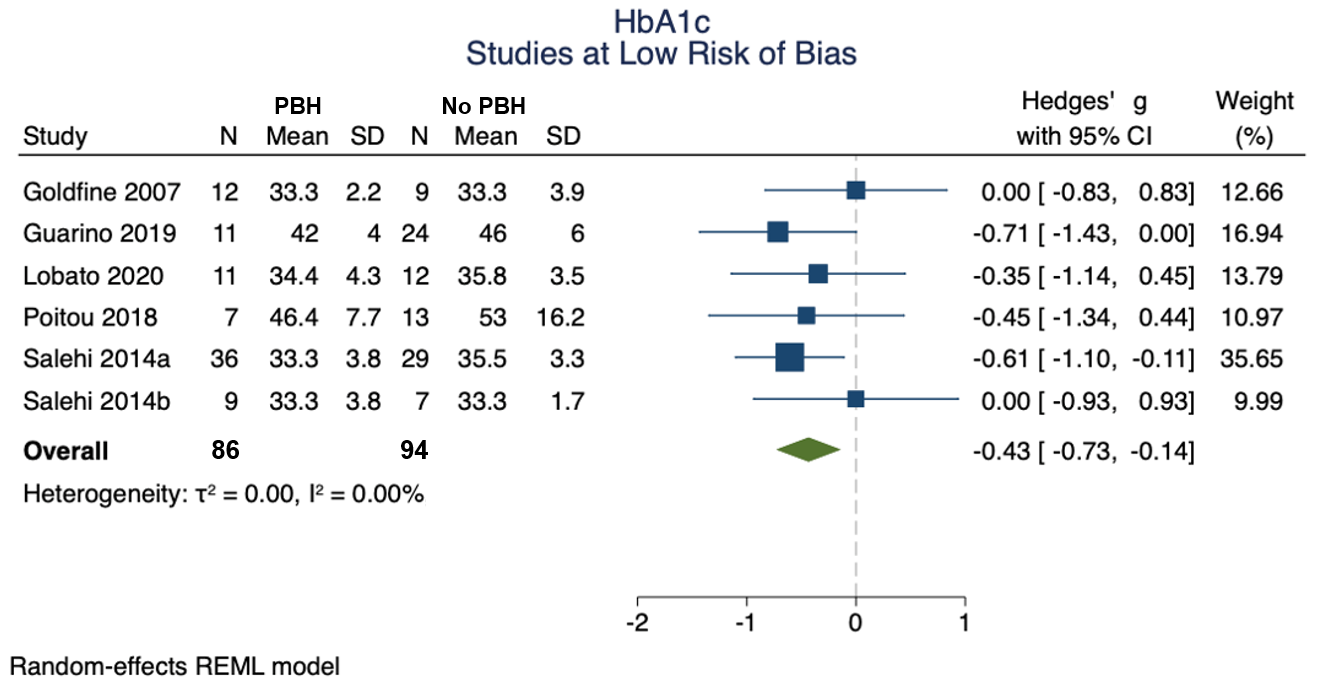

Supplement: Supplementary file 2 — Supplementary Material 2 [file 11154_2023_9823_MOESM2_ESM.docx]
